# Will someone knock on my door? COVID-19 and social work education

**DOI:** 10.1177/1473325020981075

**Published:** 2021-03

**Authors:** Bharati Sethi

**Affiliations:** School of Social Work, King’s University College, Western University, London, Canada

**Keywords:** Reflection, social justice, social work education

## Abstract

Guided by a person-in-environment framework and aspirations to advance social justice, the social work profession is concerned with intervening at the individual and society level. In this essay, the author reflects on individualism-collectivism, loneliness, and community belonging in the context of her lived experiences and the COVID-19 outbreak. She maintains that the micro-macro fragmentation is problematic to social work's quest for social justice. Social work must examine the place of ‘community practice' in its professional curriculum to equip students with tools to fully comprehend the changing and increasingly complex social workers' role.

I have lived in London, Ontario, for five years. As an immigrant, this is my third internal migration and one that I find the most challenging. Having no family in Canada once again, I had to leave behind my friends, support network, and the community I had built in Brantford, Ontario. As a single woman in her 40's, I have struggled to re-build my social capital in this city. Growing up in India, I experienced a strong sense of the collective that fostered interconnectedness between neighbours, families, and communities. However, when I returned to India after residing in Canada for 14 years, India I hold dear to my heart had changed. Households were smaller, and people valued personal independence over interdependence and family relationships. I could barely recognize the beautiful suburb in Mumbai, where I spent years playing ‘sticks and stones' with neighbours' children and friends. The pace of life was much faster than I remember. Individualistic practices and values were unmistakably alive in Mumbai and other large cities. While individualistic cultures place a higher value on the self (e.g. individual goals, personal freedom and rights, and independence), in collectivistic cultures, a sense of identity is shaped within a social and cultural context, and such cultures embrace community goals and interdependence (Santos et al., 2017).

The authors (Santos et al., 2017) confirm that the rise in individualism is not restricted to developed or industrialized nations. Based on an analysis of 51 years' worth of data detailing across 78 countries, their study found a “global trend towards individualism” (1229). As a social worker, I wrestle with the question: *Is there a link between individualism and loneliness* that Hendrix (2018) hints at in the national review article, *Lonely America*. He observed that loneliness increased by 20% to 40% in America since the 1980s.

Even though my memories of India were those of belonging and social connectedness, we cannot assume that collectivist countries have fewer lonely individuals than states that identify as individualists. Growing up in a collectivist country, I often felt isolated and disengaged from my neighbourhood and the Hindu community. It is possible that despite being surrounded by family, the experience of loneliness was due to the moral, religious, and social expectations placed upon my gender by a patriarchal society. Despite the presence of strong family ties, when cultural expectations and political contexts dictate women's lives, it may limit her access to meaningful friendships and other social relationships, contributing to loneliness (Al Khatib, 2012).

This rise in loneliness appears to be most prevalent amongst adults who are “unmarried and the uncommitted to the community” Hendrix (2018, para. 2). However, the interaction of marital status and the strength of community bonds in predicting loneliness reveals mixed findings. On the one hand, Heu et al. (2019: 788) found that “loneliness was higher among individuals who lived alone and had weaker community bonds.” On the other hand, these authors (Heu et al., 2019: 788) also proposed that “average loneliness was higher in regions with low shares of individuals living alone and stronger community bond.” The point is that individualism/collectivism is multifaceted constructs. Political, economic, and social factors intersect to shape an individual and communities' experiences. At the personal level, it is worth considering the importance of an individual's perception of the self or others as embedded within one's social environment as a significant factor contributing to loneliness (Heu et al., 2019). Displacement and loss of social capital (such as immigrants' social networks and changes in family relationships) can influence the experience of loneliness (Al Khatib, 2012). For instance, as a woman with an East Indian background, I often experienced multiple aggressions related to my race, ethnicity, immigration status, and gender. Consequently, anticipating rejection (Al Khatib, 2012), I relied on myself rather than reaching out to the broader community to cope with stressors. More than loneliness, social isolation resulting from the feeling of disconnectedness from the immigrant and local Canadian community paralyzes me. Let me explain.

A decade later, the incident of a dead man and his dog still haunts me. This man lived in a neighbourhood very much like mine with houses close to one other. It was not until a concerned friend alerted the police after returning from a two-week vacation that they found his dead body with his dog next to him. The dog was barely alive. She found a new home with a loving family who heard about the incident. I wondered if any of the neighbours heard the dog barking for days? Were the neighbours curious about the piles of newspapers and flyers collected on his front porch? Did they miss him when he did not pass by their home on his routine walk with his dog? Did anyone knock on his door? My constant fear is that no one would knock on my door if I died unexpectedly. The concern is not for me; instead, it is for the safety of my two cats. I am not alone in my experience of isolation from the mainstream community. The loneliness minister's appointment has done little to address chronic loneliness in the U.K. (Birnstengel, 2020). Loneliness and social isolation are associated with multiple adverse mental and physical health problems (Heu et al., 2019).

“What is the opposite of loneliness? Is it belonging?” asks Enayati (2012), in a CNN health special, *The importance of belonging.* The COVID-19 has highlighted the significance of fostering inclusion and community connections to reduce social suffering. What were we grieving for most during this pandemic? What were we longing for as we navigated the challenging time? Amidst the panic of toilet paper shortage and disinfecting wipes, the cries around the world that I heard were about ‘social distancing.' While we recognized the urgency of stay-at-home orders to curtail the spread of the virus, most of us missed the face-to-face contact with colleagues, family, and friends.

For me, the quarantine stirred up painful emotions of loss and added more pain to my experience of social isolation. As a waitress for almost 14 years, I can still recall the mad rush on Mother's Day. As I walked down the local Richmond street in May 2020, the roads usually buzzing with customers celebrating their moms was now almost empty. Like the disruption of celebrations, the pandemic disturbed mourning, thus, magnifying people's grief. People could not gather to celebrate the lives of their loved ones. For many immigrants, the situation was even worse. A friend lost her father-in-law unexpectedly during his visit to Canada. She relied on her transnational supports to help her navigate the complex and challenging COVID-19 situation. In the absence of traditional mourning rituals, she struggled to say good-bye and find closure.

Older adults were at the mercy of the pandemic. As care homes made efforts to contain the coronavirus's spread, I could not visit my 96-year old friend in Brantford. I tried to comfort her during our phone conversations, but I could do little to help her through the challenges to adapt to the new normal of participating in the live church sermons online. Above all, she missed the fellowship of Sunday mass at the church, her pillar of strength.

While the internet and online platforms (such as Facebook, Zoom, WhatsApp, and Webex) have changed how we communicate, the pandemic reiterated that these online social networks/media are a poor substitute for face-to-face social connections. I witnessed creative and innovative ways in which people formed communities that never existed before the coronavirus outbreak. One such example is of people connecting virtually to make face masks in Canada and worldwide. My friend Rose made over 500 cloth masks for front line workers. The local temple and other faith-based organizations recruited volunteers as part of the 'grocery shopping community' that assists older immigrants in staying healthy. It is noteworthy that while most people did rise to the challenge, much of the help came from community-based organizations. These organizations played a critical role in caring for and coordinating localized short and long-term community efforts to respond to the most vulnerable's acute needs.

Kindness and heroism took flight during the COVID-19 pandemic. As a nation, we celebrated the courage of health care and other essential workers and first responders, who put themselves at risk every day. Sadly, amongst the heroism and altruism, I was disturbed by the dramatic increase in racially motivated physical and verbal violence directed at people of Chinese and Asian descent (especially women) (Canadian Association for Social Work Education (CASWE-ACFTS), 2020) and the “pubic scolding or social media shaming” (Samuel, 2020, para 4) of “covidiots” (Samuel, 2020, para 2) amid COVID-19 outbreak.

These events and issues I discussed above raise an important question: *What has the COVID-19 pandemic to do with social work education and practice?* I am one of the few instructors who teach the macro-level courses such as *Community Practice*, in a two-year ‘Direct Practice (non-thesis) program' to Master of Social Work students. Despite faculty and field placement team's efforts to emphasize the importance of micro (individual, family, and group) and macro (organization, community, and government) integration in social work practice, some students disregard the value of a community or policy class in their aspiration to be clinical practitioners. In my teaching evaluations, I have received student comments suggesting that a mandatory community class is a ‘waste of time' in a direct practice program.' My colleagues with whom I collaborate in multi-site community-engaged research projects share my concern about the influence of the ‘clinical turn' on the social work profession. I am not against a direct practice curriculum. *Community practice is also direct practice*. Despite the social work profession's longstanding commitment to further social justice, the lack of individual-social dimension integration reflects the “professionalization of social work, marked by the striving for recognition and prestige, has fostered conservatism and identification with the status quo” (Weiss-Gal, 2008: 73).

The pandemic crisis has exposed the oppression of those who live in society's margins due to their gender, ability, class, age, or immigration status. The government has committed millions of dollars to help vulnerable individuals and families to get back on their feet. However, neoliberalism that supports laissez-faire economics and capitalism has weakened the welfare state. Such neo-liberal philosophy undermines social equality and ultimately puts the burden of wellbeing on the individual rather than the government.

Post-COVID crisis, I wonder if the students will reflect upon the significance of community capacity building and policy practice to ‘clinical' work? In the London Free Press interview (Rivers, 2020: A4), Arundel, the coordinator of field education at the School of Social Work, King's University College, Western University, explained how students completed their field practicum during the pandemic. She noted: “Social workers get in the trenches, and we respond in a crisis.” These trenches included hospitals, community agencies, family health teams, employment agencies, disability services, and justice sectors. Elaborating further, Arundel stated: “to shut down with this crisis wouldn't be consistent with social work values” (Rivers, 2020: A4).

As the spread of COVID-19 across the world continues to create economic, social, and political havoc, this crisis also provides an opportunity for social workers to reflect on our professional trend to popularize the individual service user at the micro, mezzo, and macro level. We must face the fact that the pandemic crisis has dramatically changed communities and how people live, work, and interact with one another. That means post-COVID, the role of social workers as change agents, have become even more complicated. Therefore, it is crucial to reflect upon the ‘coverage' given in the social work curriculum to the macro social environment, such as inequality and inequity created due to oppressive social policies, political, and economic structures. At worst, students graduate with a minimum foundational knowledge of community practice. At best, there is a consensus concerning social work integrative practice in the curriculum and teaching (Taylor et al., 2005). The interaction of micro-courses with the macro and macro components with the micro is indispensable to students' understanding of the complexity of human behaviour within their environment and the dignity and sustainability of our profession.

Even though the pandemic concerns *everyone* and *all* countries, people of colour are more vulnerable than the white population to the virus and its economic, social, and health consequences based on their race and ethnicity (Ro, 2020). It is, therefore, important that the social work curriculum promotes “an understanding of the complex macro social environment, in which globalization, privatization, immigration, and other major social forces shape the constellation of social, political, and economic relations in small towns, major metropolitan areas, and nations alike.” (Taylor et al., 2005: 73). The inclusion of “ diversity and difference in practice” as one of the Core Competencies for Baccalaureate and Master's Social Work Programs (Council on Social Work Education, 2020: 7) is a step in the positive direction to recognize and address racism at the personal, organization, cultural, and societal levels. Still, holistic and culturally responsive interventions are needed to respond to the demographic reality and how the pandemic has changed the way we practice. Such competence necessitates problematizing the compartmentalization of intervention strategies in social work education and practice (Greene and Schriver, 2016; Weiss-Gal, 2008).

It is mostly at the individual level that social workers address loneliness. COVID-19 has raised the urgency to foster embeddedness and collectivism at the community level (Heu et al., 2019) and the nation. “In an era of hyper-individualism, we should rekindle the bonds of the community” to promote the resilience of individuals, families, organizations, and communities (Hendrix, 2018, para 1). As I bring this narrative to a close, I signed up for the neighbourhood hub born during COVID-19 out of our innate desire for human connections during this momentous crisis. *There is hope. Someone will knock on my door!*
